# Dedifferentiated endometrioid adenocarcinoma with trophoblastic components and elevated serum alfa-fetoprotein: A case report and literature review

**DOI:** 10.1097/MD.0000000000010551

**Published:** 2018-04-27

**Authors:** He Cai, Rong Zhou, Wanying Liang, Jianliu Wang

**Affiliations:** Obstetrics and Gynecology Department, Peking University People's Hospital, Beijing, China.

**Keywords:** dedifferentiated endometrioid carcinoma, metastases, trophoblastic differentiation, undifferentiated carcinoma

## Abstract

**Rationale:**

Dedifferentiated endometrioid adenocarcinoma (DEAC) consisted of a combination of undifferentiated and differentiated carcinoma is more aggressive than other conventional endometrioid adenocarcinomas.

**Patient concerns:**

A 33-year-old woman with atypical vaginal bleeding was refereed to our hospital. She had an endometrial biopsy in a local clinic which showed differentiated endometrioid carcinoma with trophoblastic components. High levels of β-Human chorionic gonadotropin (β-hCG) and alfa-fetoprotein (AFP) were detected in the patient's serum.

**Interventions:**

The patient underwent total hysterectomy with bilateral salpingo-oophorectomy, total omentectomy and systemic pelvic lymphadenectomy in our center.

**Diagnosis:**

Pathological investigation indicated that the tumor had well differentiated and undifferentiated adenocarcinoma as well as trophoblastic components.

**Outcomes:**

Serum β-hCG and AFP dropped significantly after operation. But three weeks later, the patient had developed pulmonary metastases and elevation of serum β-hCG. She died of the disease five months after surgery.

**Lessons:**

DEAC with trophoblastic differentiation seems to follow an aggressive course with early metastasis and poor clinical prognosis. However, due to small number of cases, further studies are necessary.

## 1 Introduction

Choriocarcinoma can be classified into gestational and nongestational (germ cell) types. It was characterized by a distinctive admixture of cytotrophablasts, intermediate trophoblasts, and syncytiotrophoblast, the latter accounting for the elevation of serum human chorionic gonadotropin (β-hCG). Previous studies have reported that nongerm cell neoplasms may display trophoblastic differentiation, most commonly in the form of admixed components of choriocarcinoma.^[1,2]^ In addition, several case reports have shown that elevated serum level of β-hCG was detected in patients with gynecologic tract cancers, including in 10% to 38% of patients with ovarian, cervical cancers, and endometroid carcinoma.^[2]^

Dedifferentiated endometrioid adenocarcinoma (DEAC) of the uterus, which consisted of a combination of undifferentiated and differentiated carcinomas, was first described by Silva et al in 2006.^[3]^ DEAC confers more aggressive malignant behavior than other conventional endometrioid adenocarcinomas.^[3]^ DEAC can represent a diagnostic challenge due to the lack of awareness of this entity combined with its low incidence and misdiagnosis.^[4]^

To date, trophoblastic components in DEAC have rarely been reported. In this study, we report a young woman of DEAC with trophoblastic differentiation as well as elevated levels of serum β-hCG and alfa-fetoprotein (AFP). Both the patient and her father were well-informed about this study and a signed consent was obtained. The study was approved by the local ethics committee of Peking University People's Hospital.

## 2 Case report

A 33-year-old woman, G_0_P_0_, who complained of atypical vaginal bleeding and abdominal pain during a period of 4 months was referred from a local clinic. The patient was 165 cm in height, weighed 100 kg, and had a body mass index of 36.7 kg/m^2^. Her past medical history included schizophrenia treated with risperidone and an appendectomy at the age of 8. An endometrial biopsy revealed differentiated endometrioid carcinoma with trophoblastic components. Then, she was referred to our hospital. Physical examination revealed the presence of a solid mass measuring 8 cm in the left ovary and anther tumor mass in the uterus. Transvaginal ultrasonography showed a mixed echogenic mass in the left ovarian, size 138 x 90 x 65 mm; a mass invading the uterine, size 64 x 54 x 53 mm. Magnetic resonance imaging (MRI) of the pelvic revealed an ill-defined large heterogeneous soft tissue masses in the uterus and left adnexa area. Preoperative chest computed tomographic (CT) scan was negative. Because of the histologic and imaging results, both of the serum β-hCG and AFP were tested and found to be elevated (β-hCG: 238,418.35 U/L, normal value ≤ 4 U/L; AFP: 800.10 ng/mL, normal value ≤ 7 ng/mL). Other markers including carbohydrate antigen (CA)-125 and neuron-specific enolase (NSE) were within the normal range. She underwent abdominal surgical treatment, and the left ovary and fallopian tube were removed and sent for intraoperative consultation. On frozen section, a poorly differentiated ovarian malignant tumor was diagnosed. Then, total hysterectomy with contralateral salpongo-oophorectomy, total omentectomy, and systemic pelvic lymphadenectomy was performed.

Gross morphologically, the uterus measured 16 x 10 x 5 cm. Two protruding masses were found in the endometrial cavity. Both of the 2 polypoid masses (size 6 x 5 x 5 cm and 2 x 1 x 1 cm, respectively) were infiltrating into the myometrium. And the smaller one was extending from the fundus to the cervix. The left ovarian tumor (12 x 10 x 6 cm) was involved with large necrosis and hemorrhages.

**Figure 1:**
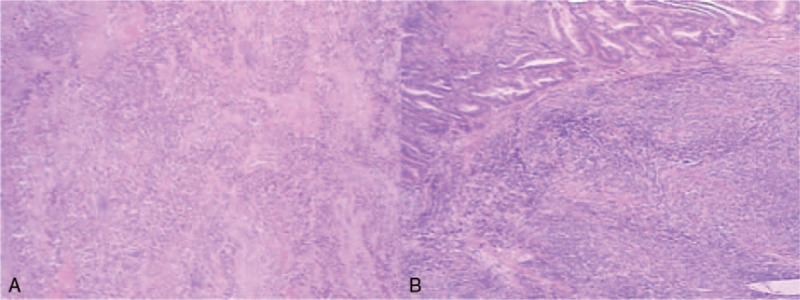
(A,B) Histopathologic findings of dedifferentiated endometrioid adenocarcinoma. (A) The majority of the undifferentiated carcinoma was found in the endometrium and deeper myometrium. (B) Undifferentiated carcinoma was located in the left ovary (lower right).

**Figure 2:**
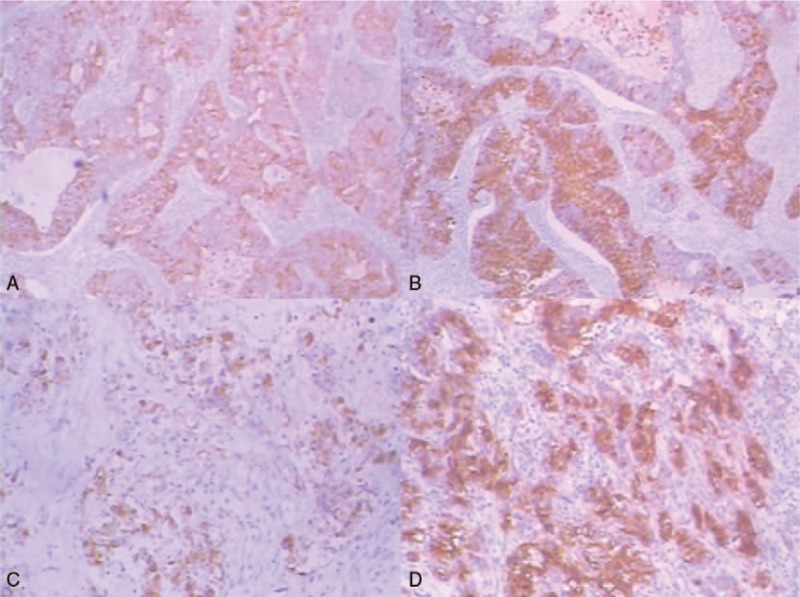
(A–D) Immunohistochemical findings of dedifferentiated endometrioid adenocarcinoma. (A) Tumors showed focal positive staining for CK7 (x40). (B) Tumors stained strongly for PLAP (x40). (C) Tumor cells were strongly positive for EMA (x100). (D) Expression of PLAP (x100).

Microscopically, both of the 2 masses in the uterus were composed predominately of intermediated-sized, poorly differentiated cells without obvious nested or trabecular patterns. The tumors had deeply (> 50%) infiltrated the myometrium and showed extensive necrosis (Fig. 1A). In addition, multinucleated choriocarcinoma-like cells were identified in the area of confluent necrosis. Involvement of undifferentiated carcinoma (UC) components was also identified in the left ovary (Fig. 1B). However, fewer syncytiotrophoblasts were presented in ovarian lesion than that in the uterine lesion. Lymphovasular invasion was found. The tumor was diagnosed as DEAC metastatic to the left ovary. According to the International Federation of Gynecology and Obstetric (FIGO) system, the case was considered FIGO stage IIIA. A panel of immunohistochemical (ICH) analysis was performed to confirm the histologic diagnosis (Fig. 2A–D). The results of ICH analysis are summarized in Table 1.^[2,5]^ In the uterus, focal positivity for cytokeratine (CK7), epithelia membrane antigen (EMA), and vimentin was detected in the UC components suggesting epithelia neoplasms. Markers such as β-hCG, human placental lactogen (HPL), and alkaline placental phosphatase (PLAP) were positive in the multinucleated choriocarcinoma-like cells. PAX8, estrogen receptor (ER), and progesterone receptor (PR) staining were absent in UC components. And, AFP and CD30 status were both negative.

**Table 1:**
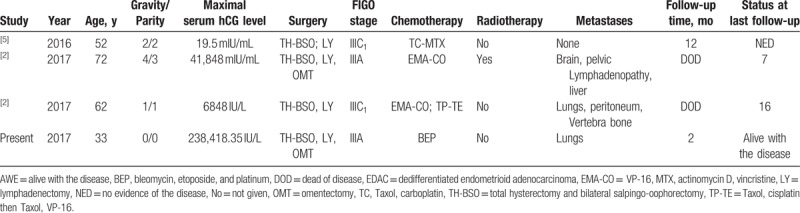
Cases of EDAC with trophoblastic differentiation in the literature.

**Figure 3:**
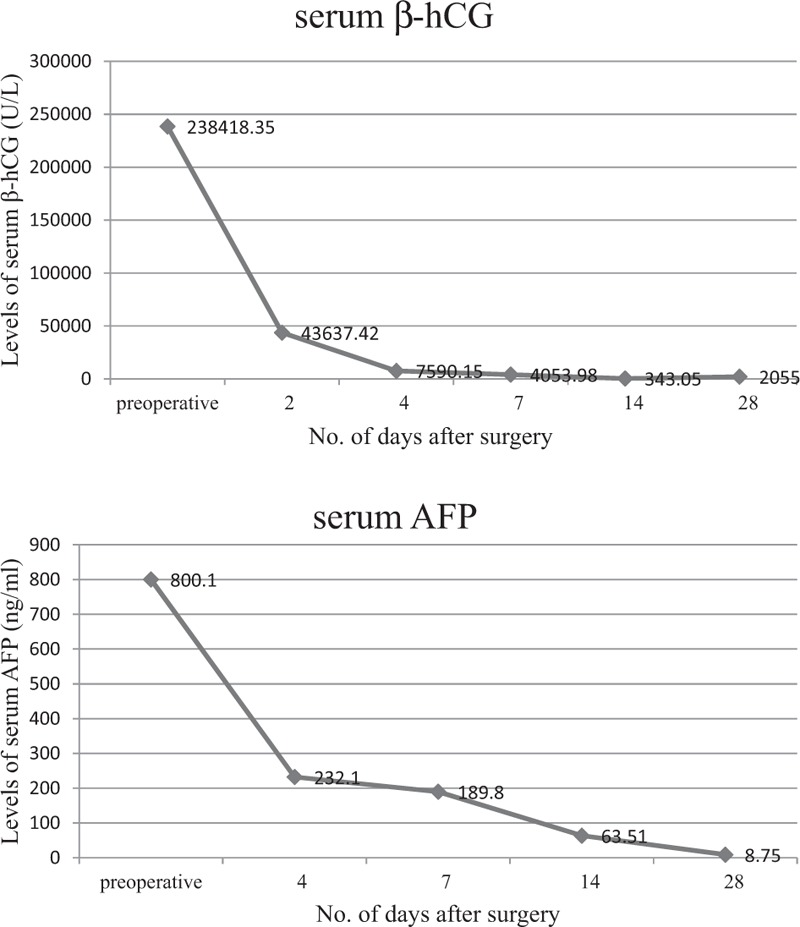
Concentrations of serum β-hCG and AFP.

Both the serum β-hCG and AFP were dramatically decreased postoperatively (Fig. 3). Unfortunately, 3 weeks postoperatively, nodules were identified in the lungs by chest CT, with associated elevations in her serum β-hCG. No adjuvant chemotherapy was given since the patient declined. She was dead of extensive disease approximately 5 months after her primary surgery.

## 3 Discussion

The World Health Organization (WHO) classification defines DEAC as a malignant tumor with an epithelial structure that is too poorly differentiated to be placed in any other category of carcinomas.^[6]^

DEAC is characterized by the coexistence of grade 1/2 endometrioid carcinoma and UC with solid sheets of medium-sized monotonous epithelia cells. Reviewing cases of endometrial adenocarcinomas, Silva et al^[3]^ found that cases of UC represented 9% of endometrial carcinomas. Seventy-one percent of cases of UC were admixed with low-grade endometrioid adenocarcinoma referred to as DEAC.^[3]^

There is no definite percentage of the UC component needed in the neoplasm for the diagnosis of DEAC, but the presence of even a small undifferentiated component appears to be associated with poor clinical outcomes.^[7–9]^ In our case, despite the timely surgery, the patient developed pulmonary metastases 3 weeks after the surgery.

Here, we reported a case of DEAC with trophoblastic differentiation as well as elevated serum AFP level. To our knowledge, this has never been reported before. Cases of tumors exhibiting trophoblastic components without fertilized ova as precursors have been reported in other organs.^[10,11]^ Garcia and Ghali ^[11]^ had previously reported the case of gastric carcinoma with both trophoblastic and yolk sac component. Endometrioid carcinoma with trophoblastic differentiation is a rare entity with only a small numbers of studies reported. Searching for cases of endometrial carcinoma with trophoblastic components, a total of 29 cases were identified.^[2,12]^ All cases presented with elevated serum or urinary β-hCG before or shortly after treatment, except 1 case with normal serum β-hCG first measured after histologic diagnosis.^[12]^ It means that extra-embryonal differentiation may manifest itself biochemically with β-hCG production and morphologically with choricocarcinoma component.^[13]^ In the present case, trophoblastic components were identified under the microscope and a dramatically elevated serum β-hCG level was detected before surgery.

The histogenetic mechanism for trophoblastic components in endometrial carcinomas remains controversial. A gestational basis is unlikely, as our patient was nulligravid. More importantly, against the gestational basis is the fact that choriocarcinomatous differentiation is recognized in carcinomas arising from nongynecolgic sites.^[14]^ Several studies suggested areas of trophoblast-like cells, probably derived through metaplasia of the tumor cells.^[13]^ By morphologic and molecular genetic analysis, Olson et al suggested that there is clonal evolution from endometrioid carcinoma to trophoblastic tumor.^[15]^ A recent study also indicated that the common genetic changes between the trophoblastic and non-trophoblastic components are evidence of clonal origin. The former one also displayed additional alterations not found in the latter one, suggesting trophoblastic areas evolved from the endometrioid carcinoma.^[16]^

Previous studies have shown that the most frequent associated histotype of carcinoma with trophoblastic differentiation was endometrioid carcinoma, which was seen in one-third of the total cases. Adding our case, only 4 cases were described as DEAC (Table 1).^[2,5]^ These 3 patients previously reported were relatively older (age range 52–72 years). Unlike them, this patient was young and had no history of pregnancy. All patients underwent surgical removal of uterus, bilateral adnexa, as well as pelvic lymph nodes as the primary surgical management. Patient in this study declined the chemical therapy, and other patients previously reported all received adjuvant chemotherapy. Except case 1, other patients were presented with metastasis postoperatively. Sites for metastases included lungs, brain, bone, peritoneum, and liver.

However, due to small number of cases, it is too early to conclude that the carcinomas with trophoblastic differentiation follow an aggressive course with early metastasis and poor clinical prognosis. Intrinsic biologic aggressiveness of the coexisting carcinoma, FIGO stage at presentation, patient age, the responsiveness to adjuvant treatments, and other unknown factors are potential confounding variables.

There was 1 intriguing finding in our case. The patient had elevated AFP throughout the entire medical course, although no histologic evidence of yolk sac tumor-like components was noted. However, the decrease in AFP after surgery excluded the false-positive possibility by the testing method used. The possibility of yolk sac tumor or other germ cell tumors were excluded by ICH staining. Because variable epithelia makers, including vimentin, keratins, and EMA, were expressed in regions, whereas neither AFP nor CD30 was positive. To explain the finding, further investigations are needed.

## Acknowledgment

We thank Professor Danhua Shen and doctor Limin Zhai for providing pathological materials included in the study.

## Author contributions

**Conceptualization:** Rong Zhou.

**Funding acquisition:** Jianliu Wang.

**Resources:** Rong Zhou.

**Supervision:** Jianliu Wang.

**Writing – original draft:** He Cai.

**Writing – review & editing:** Wangying Liang.
